# Supplementary material for: Resolving the full spectrum of human genome variation using Linked-Reads
Source: Genome Res. 2019 Apr;29(4):635–45. doi: 10.1101/gr.234443.118 (PMC6442396; doi:10.1101/gr.234443.118)
Supplement: Supplemental Material [file supp_gr.234443.118_Supplemental_longranger-2.2.zip › longranger-2.2.tar.bz2!/lr2.2-public/lib/stan/cmdstan/stan/doc/api/html/namespacemembers_func_f.html]

Stan: Namespace Members


|  |  |
| --- | --- |
|  | Stan  2.5.0  probability, sampling & optimization |


- Main Page
- Related Pages
- Namespaces
- Classes
- Files

- Namespace List
- Namespace Members

- All
- Functions
- Variables
- Typedefs
- Enumerations
- Enumerator

- a
- b
- c
- d
- e
- f
- g
- h
- i
- j
- l
- m
- n
- o
- p
- q
- r
- s
- t
- u
- v
- w


All Classes Namespaces Files Functions Variables Typedefs Enumerations Enumerator Friends Macros Pages

### - f -

- F32()
  : stan::math
- fabs()
  : stan::agrad
- factor\_cov\_matrix()
  : stan::prob
- factor\_U()
  : stan::prob
- falling\_factorial()
  : stan::agrad
  , stan::math
- fdim()
  : stan::agrad
  , stan::math
- fill()
  : stan::math
- finite\_diff\_grad()
  : stan::model
- floor()
  : stan::agrad
- fma()
  : stan::agrad
- fmax()
  : stan::agrad
- fmin()
  : stan::agrad
- fmod()
  : stan::agrad
- fpclassify()
  : boost::math
- frechet\_ccdf\_log()
  : stan::prob
- frechet\_cdf()
  : stan::prob
- frechet\_cdf\_log()
  : stan::prob
- frechet\_log()
  : stan::prob
- frechet\_rng()
  : stan::prob
- fun\_scalar\_type()
  : stan::gm


---

[ Stan Home Page ]

*© 2011–2014,
Stan Development Team.*
